# RNA Interference: A Promising Approach for the Treatment of Viral Hepatitis

**DOI:** 10.5812/kowsar.1735143X.812

**Published:** 2012-01-20

**Authors:** Mahsa Motavaf, Seyed Moayed Alavian

**Affiliations:** 1 Baqiyatallah Research Center for Gastroenterology and Liver Diseases, Baqiyatallah University of Medical Sciences, Tehran, IR Iran; 2 Tehran Hepatitis Center, Tehran, IR Iran

**Keywords:** RNA, Viral, Hepatitis, Human, Therapeutic

Hepatitis viruses are the leading cause of liver cirrhosis and hepatocellular carcinoma (HCC) worldwide. The hepatitis C virus (HCV), together with the hepatitis B virus (HBV), accounts for 75% of all cases of liver disease around the world, with chronic hepatitis, cirrhosis, and HCC causing 500,000 to 1.2 million deaths per year [1][2][3]. More than 170 million people worldwide have chronic HCV infection. According to the 2002 WHO report, chronic liver diseases were responsible for 1.4 million deaths, including 796,000 deaths due to cirrhosis and 616,000 deaths due to primary liver cancer. At least 20% of these deaths (more than 280,000 deaths) are probably attributable to HCV infection [4]. With the WHO officially recognizing hepatitis as a global health issue, more research needs to be conducted to develop new therapeutic interventions. Current antiviral therapies for chronic viral hepatitis are effective only in approximately half of the patients [5]. The most widely available agents for the treatment of chronic hepatitis are interferon-α (IFN-α) and nucleoside analogs such as lamivudine or adefovir [6][7]. However, treatment with these agents has some disadvantages, including possible serious adverse effects in the case of interferon treatment or recurrence of viremia after discontinuation of therapy and development of resistant mutants after prolonged lamivudine treatment [8][9][10][11]. Moreover, nucleoside analogs such as 3TC-lamivudine only interfere with viral replication and do not induce cessation of the process. The low efficacy of these agents, their adverse effects, and development of resistant viral mutations are major impediments to the clinical application of these agents for the treatment of viral hepatitis [6]. These shortcomings necessitate the development of alternative treatment strategies. Sequence-specific gene silencing using RNA interference (RNAi) is a Nobel prize-winning technology that represents a promising new approach to overcome viral infections [5][12][13][14]. RNA interference is an evolutionary mechanism for protecting the genome against invasion by mobile genetic elements such as transposons and viruses [6]. It is a process by which small interfering RNA (siRNA) with specific target sequences induce silencing of homologous genes by binding to their complementary mRNA and inducing the elimination of the mRNA molecule [15]. This process occurs post-transcriptionally in the cytoplasm and is mediated by small RNA molecules (21 to 25 nucleotides in length) that bind to their complementary mRNA targets and silence the expression of these targets [6]. The phenomenon of RNA interference (RNAi) was first described by Fire et al. They observed that in the nematode Caenorhabditis elegans, the presence of double-stranded RNA (dsRNA) resulted in sequence-specific gene silencing at the post-transcriptional level. The RNAi pathway has since been recognized as a conserved biological pathway, and several experimental models have contributed to the understanding of this process [15][16].

Subsequent to the discovery of RNAi pathways and their ability to silence a specific gene sequence, researchers have proposed that this natural protection response might be used for therapeutic purposes. The ability to achieve potent knockdown of a gene of interest with high sequence specificity makes RNAi a powerful tool for treating a variety of diseases. Since RNAi, as a natural mechanism of defense, has an antiviral effect in plants and mammalian cells, pathogenic human viruses were considered a good starting point for evaluating the therapeutic potential of RNAi [17][18][19][20]. Recently, several reports have demonstrated the use of RNAi for weakening of viral infection and replication in cultured cells. For instance, several studies have shown its antiviral effects against HIV and HBV and HCV [6][12][21][22][23][24][25].

The in vivo efficacy of RNAi against a virus was first demonstrated by McCaffrey et al. In their study, they co-delivered an HBV replicon and an expression unit encoding an anti-HBV RNAi in mice. Their results showed that a significant knockdown (99%) of the HBV core antigen in hepatocytes could be achieved by the RNAi mechanism, providing an important proof of principle for future antiviral applications of RNAi in the liver.[12] In another study designed by Amir Shlomai and Yosef Shaul, in order to evaluate the anti-HBV therapeutic potential of RNAi, the levels of viral proteins and transcripts as well as the viral replicative forms were analyzed. The results showed that RNAi is an efficient approach for reducing the level of HBV transcripts and proteins and for suppressing HBV replication [26]. Many other studies have shown that co-transfection of RNAi molecules targeting specific sequences in the HBV or HCV genome results in a significant reduction in the corresponding levels of viral transcripts and proteins. This reduction is highly selective, because only the homologous transcripts and proteins were eliminated [8][13]. Thus, RNAi-based therapies have a number of inherent and fundamental benefits [8][27].
